# Technological Factors Affecting Biogenic Amine Content in Foods: A Review

**DOI:** 10.3389/fmicb.2016.01218

**Published:** 2016-08-12

**Authors:** Fausto Gardini, Yesim Özogul, Giovanna Suzzi, Giulia Tabanelli, Fatih Özogul

**Affiliations:** ^1^Centro Interdipartimentale di Ricerca Industriale Agroalimentare, Università degli Studi di BolognaCesena, Italy; ^2^Dipartimento di Scienze e Tecnologie Agro-alimentari, Università degli Studi di BolognaCesena, Italy; ^3^Department of Seafood Processing Technology, Faculty of Fisheries, Çukurova UniversityAdana, Turkey; ^4^Faculty of BioScience and Technology for Food, Agriculture and Environment, University of TeramoMosciano Sant’Angelo, Italy

**Keywords:** biogenic amines, fermented foods, lactic acid bacteria, decarboxylase activity, a_w_, pH, temperature

## Abstract

Biogenic amines (BAs) are molecules, which can be present in foods and, due to their toxicity, can cause adverse effects on the consumers. BAs are generally produced by microbial decarboxylation of amino acids in food products. The most significant BAs occurring in foods are histamine, tyramine, putrescine, cadaverine, tryptamine, 2-phenylethylamine, spermine, spermidine, and agmatine. The importance of preventing the excessive accumulation of BAs in foods is related to their impact on human health and food quality. Quality criteria in connection with the presence of BAs in food and food products are necessary from a toxicological point of view. This is particularly important in fermented foods in which the massive microbial proliferation required for obtaining specific products is often relater with BAs accumulation. In this review, up-to-date information and recent discoveries about technological factors affecting BA content in foods are reviewed. Specifically, BA forming-microorganism and decarboxylation activity, genetic and metabolic organization of decarboxylases, risk associated to BAs (histamine, tyramine toxicity, and other BAs), environmental factors influencing BA formation (temperature, salt concentration, and pH). In addition, the technological factors for controlling BA production (use of starter culture, technological additives, effects of packaging, other non-thermal treatments, metabolizing BA by microorganisms, effects of pressure treatments on BA formation and antimicrobial substances) are addressed.

## Introduction

Biogenic amines (BAs) are organic bases, which can be present in foods and can cause several adverse reaction in the consumers. They are produced by microorganisms (mainly bacteria) through the action of decarboxylases (carboxy-lyases EC number 4.1.1.1.), which act selectively on specific amino acids in which they remove the carboxyl group with the formation of the correspondent amine and CO_2_.

In relation to their amounts and their toxicological effects, the most important BAs in foods are histamine (an heterocyclic amine deriving from histidine), tyramine and 2-phenylethylamine (deriving from the aromatic amino acids tyrosine and phenylalanine, respectively), tryptamine (heterocyclic BA from tryptophan), putrescine (a polyamine obtained through a direct decarboxylation of ornithine or through the agmatine deiminase pathway, which follow the decarboxylation of arginine to agmatine), and cadaverine (a polyamine derived from lysine; Silla Santos, 1996; Landete et al., 2008a; Tanaka et al., 2008; Marcobal et al., 2012; Wunderlichová et al., 2014). In addition, other polyamines (spermine and spermidine) can be produced with a more complex pathway, which starts from putrescine (Bardócz, 2005; Kalač and Krausová, 2005).

**FIGURE 1:**
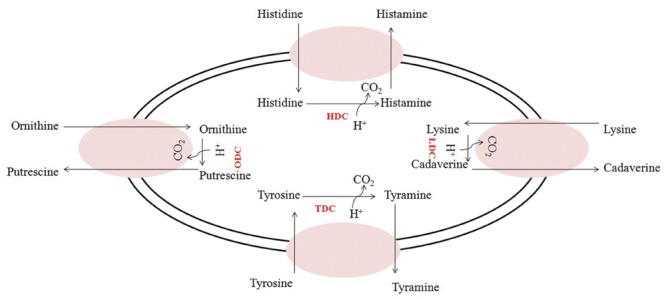
**Amino acid decarboxylation-antiporter reactions in which an amino acid is transported into the cell, where decarboxylation occurs.** A proton (H^+^) is consumed, and a carbon dioxide (CO_2_) is removed during the reaction, and the product (biogenic amines) is exported from the cell via an antiporter. HDC, histidine decarboxylase; TDC, tyrosine decarboxylase; LDC, lysine decarboxylase; ODC, ornithine decarboxylase.

There are two essential physiological reasons leading to the activation of decarboxylative pathways. From one side, decarboxylation is one of the responses of cells to acid stress and the final balance of the pathway, which consists in the loss of a carboxylic group, contributes to the intracellular (and extracellular) pH increase (Connil et al., 2002; Tanaka et al., 2008; Pereira et al., 2009; Perez et al., 2015). Furthermore, it has been demonstrated that these pathways can bring supplementary energy for the cells by energizing the protonmotive force associated to the membrane (**Figure 1**). In fact, a net positive charge is transferred outside the cell during the exchange between precursor (amino acid) and BA (Molenaar et al., 1993; Konings et al., 1997; Konings, 2006; Wolken et al., 2006; Pereira et al., 2009).

The presence of dangerous amounts of BAs is associated with a relevant growth (>7 log cfu/g) of decarboxylating microorganisms. For this reason, some authors proposed microbial quality indices based on food BA content as indirect indicators of excessive microbial proliferation (Karmas, 1981; Ruiz-Capillas and Jiménez-Colmenero, 2004; Baixas-Nogueras et al., 2005; Özogul and Özogul, 2006; Al Bulushi et al., 2009). Nevertheless, fermented foods requires (by definition) a massive growth of microorganisms which are often responsible for noteworthy BA accumulation, especially during the ripening phase (when selected starter cultures can be replaced by wild strains) or when natural (spontaneous) fermentations are adopted (Suzzi and Gardini, 2003; Ancín-Azpilicueta et al., 2008; Rabie et al., 2011a; Linares et al., 2012).

BAs can be produced both by Gram-positive and Gram-negative bacteria (Landete et al., 2008a; Marcobal et al., 2012; Wunderlichová et al., 2014). Also some fungi (yeast and molds) are involved in BA accumulation (in particular cadaverine and putrescine), but their role is debated and, for many aspects, controversial (Caruso et al., 2002; Gardini et al., 2006; Kiss et al., 2006; Tristezza et al., 2013; Qi et al., 2014).

In general, there is no legal regulation about the BA content in food. This is mainly due to the individual toxicological threshold, which can be extremely variable from few mg/kg in sensitive person to some hundred mg/kg in healthy person (Shalaby, 1996; McCabe-Sellers et al., 2006; Fogel et al., 2007; Hungerford, 2010; Knope et al., 2014). The only exception is the scombroid fish, because its richness in histidine and aptitude to support the growth of decarboxylating microorganisms, for which several national and international authorities define a limit for histamine (Anonymous, 2001; The Australian and New Zealand Food Standards Code, 2002; European Community Commission Regulation, 2005; Prester, 2011). In alcoholic beverages (wine, beer, etc.) the toxicity of BAs is enhanced by the presence of ethanol, an inhibitor of mono amino oxidases (Ancín-Azpilicueta et al., 2008). Nevertheless, the presence of high amounts of BA can be related to scarce microbiological quality of raw materials, during the improper storage of the products, as well as an uncontrolled fermentation (Suzzi and Gardini, 2003; Naila et al., 2010; Linares et al., 2012).

Recently, a qualitative risk assessment concerning BA in fermented foods was conducted in the European Union (EFSA, 2011). According to this study, histamine was present in detectable amounts in the 85% of the dairy fermented products, 83% of fermented vegetables, 45% of fermented meats, and 73% of fish products. The higher concentrations of that were found in fish sauces, dried anchovies, and cheeses. Tyramine was detected in 75% of dairy products and 83% of fish products, but the higher concentrations were found in meat product (fermented sausages and cured meats) and cheeses. Similar results were characterized in 2-phenylethylamine formation in various food products as well. In addition, putrescine, cadaverine, and tryptamine were most frequently detected in dairy food products.

This review summarizes the current state of knowledge of the effects of the major environmental and process factors on the decarboxylase activity of microorganisms. It also highlights the strategies available to reduce the BA accumulation in food products.

## Biogenic Amine Toxicity and Decarboxylating Microorganisms in Food

The decarboxylation process can be catalyzed through two biochemical pathways. The first is catalyzed by naturally occurring endogenous amino acid decarboxylases present in animal or vegetable cells and the second by exogenous enzymes produced by various microorganisms under favorable conditions (Halász et al., 1994).

Natural polyamines represent the main amines found in fresh food products (fish, fruits, vegetable, milk, and meat) where they have a physiological role associated with cell growth and proliferation (Bover-Cid et al., 2014). The intracellular biosynthesis of endogenous amines such as the polyamines spermine and spermidine and other amines like histamine involves the incorporation of aminopropyl groups into their precursor putrescine (Bardócz, 2005).

Exogenous BAs derive from decarboxylases secreted by microorganisms, which are present naturally in food products, introduced by contamination, or also added to foods as a starter culture. The enzymatic decarboxylation process depends on various factors such as the availability of the substrate in free form, the presence of decarboxylase-producing microorganisms and the medium conditions (pH, temperature, O_2_, etc.). The free amino acid are either naturally present in the food or produced via proteolysis both by endogenous proteases in the raw products and microbial enzymes (Danquah et al., 2012). In fact, proteolysis may play an important role in the release of free amino acids from tissue proteins, which offer a substrate for decarboxylases reactions (Shalaby, 1996).

### Risk Associated to Biogenic Amines in Food

The presence of BAs in food can constitute a risk to the consumer health (Gram and Dalgaard, 2002; Gram et al., 2002; Özogul et al., 2011). Ingestion of food containing high amounts of BAs is implicated in various pharmacological and toxicological reactions. In fact, BA intake can cause headaches, heart palpitations, vomiting, and diarrhea. Moreover, hypertensive crises have been reported after consuming food containing BAs, such as cheese, wine, beer, and vegetables including sauerkraut, broad bean, banana peel, and avocado (Moret et al., 2005; Maintz and Novak, 2007; Hungerford, 2010).

Once the decarboxylase enzymes are synthesized by the bacteria, BA production can continue even if the bacteria are eliminated from the food product by cooking or other technological treatment. The BAs produced are heat stable and, once formed, are not destroyed by cooking, smoking, freezing, or some other type of preservation techniques (Becker et al., 2001).

Under normal conditions in humans, exogenous amines ingested with food are rapidly detoxified (Hornero-Mendez and Garrido-Fernandez, 1997). The enzymes monoamine oxidase (MAO) and diamine oxidase play an important role in this detoxification process. However, the severity of BA toxicological effects depends on the intake with food, on individual allergy and on the consumption of MAO inhibiting drugs, alcohol, and other food amines (Silla Santos, 1996; Sathyanarayana Rao and Yeragani, 2009).

The BAs with the more severe acute effects for human health are histamine and tyramine.

Histamine causes a symptomatology known as “scombroid fish poisoning” consisting in flushing of the face, neck and upper arms, oral numbness and/or burning, metallic taste, headache, itchy rash, heart palpitations, asthma attacks, hives, gastrointestinal symptoms, and difficulties in swallowing (Lehane and Olley, 2000; Maintz and Novak, 2007; Hungerford, 2010; Knope et al., 2014). This type of food intoxication often results from consumption of scombroid fish (such as tuna, sardines, anchovies, bonito, mackerel, etc.), which are rich in histidine, because of the proliferation of histidine decarboxylative Gram-negative bacteria. However, these BAs can also be found in fermented products (wine, cheese, fish sauce, and fermented meat) where it is mainly produced by lactic acid bacteria (LAB).

Tyramine toxicity is known as “cheese reaction” because it was initially observed following the consumption of cheeses with high level of this BA (Shalaby, 1996). Tyramine has a vasoconstrictor effect and causes dietary-induced migraine, increased cardiac output, nausea, vomiting, respiratory disorders, and elevated blood glucose (Shalaby, 1996; McCabe-Sellers et al., 2006; Scheepens et al., 2010; Stadnik and Dolatowski, 2010; Marcobal et al., 2012). This increase in blood pressure due to tyramine can cause heart failure or brain hemorrhage (Naila et al., 2010). Besides to these effects, tyramine has also been determined to have an effect on the gut microbiota. The adherence of some enteropathogens, such as *Escherichia coli* O157:H7, to intestinal mucosa is increased in the presence of tyramine (Russo et al., 2012). This type of food intoxication is usually associated with the consumption of fermented foods because LAB are the most efficient producers of tyramine (Shalaby, 1996; Ladero et al., 2012).

As far as other BAs, putrescine and cadaverine have low toxicological properties on their own (Košmerl et al., 2013). However, they could potentiate the effects of histamine and tyramine toxicity by inhibiting their metabolizing enzymes (Landete et al., 2007; Al Bulushi et al., 2009). Moreover, putrescine and cadaverine can act as a precursor to the formation of carcinogenic *N*-nitrosamines in the presence of nitrite (Rauscher-Gabernig et al., 2012; De Mey et al., 2014). High concentrations of putrescine have been linked to tumor development such as promotion of CT-26 colon tumor cell growth (Farriol et al., 2001).

Despite their cellular functions, the polyamine agmatine, spermine, and spermidine catabolism and their excess levels can lead to toxicity. It has been demonstrated that spermine and spermidine could decrease blood pressure, inhibit blood clotting and provoke respiratory symptoms and neurotoxicity resulting in renal insufficiency (Pegg, 2013).

Even if 2-phenylethylamine is present naturally in several mammalian tissues such as the brain, it is found in certain foodstuffs (chocolate, cheese and red wine) and has been known to trigger migraine attacks and increase blood pressure (Panoutsopoulos et al., 2004; Souza et al., 2005).

### Main Microbial Groups Involved in BA Production in Foods

Among Gram-negative bacteria, spoilage microorganisms belonging to enterobacteria and pseudomonads are known as the major producers of histamine, cadaverine, and putrescine (Ben-Gigirey et al., 2000; de las Rivas et al., 2007; Pircher et al., 2007; Lorenzo et al., 2010). The genus *Photobacterium* is often involved in the accumulation of histamine in fish and seafood products, together with *Aeromonas hydrophila* and enterobacteria such as *Morganella morganii, Enterobacter aerogenes, Raoultella planticola*, and *Klebsiella oxytoca* (Morii and Kasama, 2004; Veciana-Nogués et al., 2004; Kanki et al., 2007; Al Bulushi et al., 2009; Fernández-No et al., 2010; Küley et al., 2013). BAs produced by enterobacteria were also found in fermented sausages, meat, and cheeses (Ruiz-Capillas and Jiménez-Colmenero, 2004; Bover-Cid et al., 2009; Linares et al., 2011).

The ability to produce BAs is widespread also among Gram-positive bacteria. The decarboxylase activity has been found in strains belonging to the genera *Staphylococcus* and *Bacillus* (Landeta et al., 2007; Chang and Chang, 2012). However, the attention has been mainly focused on LAB, which are commonly present in the ripening microbiota of several fermented foods. In fact, LAB can produce histamine, cadaverine, putrescine, but, in particular, they are the most efficient producers of tyramine (Arena and Manca de Nadra, 2001; Pereira et al., 2001; Suzzi and Gardini, 2003; Pircher et al., 2007; Buňková et al., 2009, 2011; Kuley and Özogul, 2011; Ladero et al., 2012). The tyrosine decarboxylase of LAB is often able to decarboxylate also phenylalanine (producing 2-phenylethylamine) even if with a minor efficiency compared to tyrosine (Marcobal et al., 2006a). This ability was confirmed by several authors which also underlined that phenylalanine was used as substrate by the enzyme only when tyrosine was not available (Beutling and Walter, 2002; Pessione et al., 2009; Bargossi et al., 2015b).

*Oenococcus oeni, Lactobacillus hilgardii, Lactobacillus fructivorans, Pediococcus parvulus, Lactobacillus brevis* are responsible for amines accumulation in wine. Decarboxylating strains of *Lactobacillus curvatus, Enterococcus faecalis, Enterococcus faecium, Lactobacillus fermentum, Lactococcus lactis, Streptococcus thermophilus*, and *Lactobacillus paracasei* were isolated from cheese, meat, and sausage with high BA content (Moreno-Arribas et al., 2003; Smit et al., 2008; Marcobal et al., 2012; Russo et al., 2012; Wunderlichová et al., 2014).

Yeast strains belonging to various species including *Saccharomyces cerevisiae, Hanseniaspora uvarum, Candida stellata, Kloeckera apiculata, Metschnikowia pulcherrima*, and *Brettanomyces bruxellensis* are also capable of aminogenesis (Romano et al., 2007). Yeast strains isolated from grapes and wines are able to yield high BA amounts (Caruso et al., 2002). In addition, the fungus *Botrytis cinerea* is considered a producer of BAs in grape must (Bäumlisberger et al., 2015).

The genetic organization of decarboxylase clusters has been reviewed for tyramine (Marcobal et al., 2012), histamine (Landete et al., 2008a), and putrescine (Wunderlichová et al., 2014).

## Environmental Factors Influencing Biogenic Amine Formation

The main environmental factors affecting microbial activities in foods are temperature, salt concentration, and pH. These factors can influence the formation of BAs in two ways. In first instance, they are responsible for the overall metabolism of the decarboxylating cells. In addition, the activity of decarboxylases depends on the same parameters. The optimal values of environmental factors for these two different aspects can be different (Bargossi et al., 2015a) and the final amount of BAs is the result of this double influence. In other words, growth of aminobiogenetic bacteria is an essential but not sufficient condition for BA production (Marcobal et al., 2006b). Moreover, some decarboxylase can maintain their activity independently of the integrity of the microbial cells in a wide range of conditions. This has been demonstrated for tyrosine decarboxylase in lactobacilli (Moreno-Arribas and Lonvaud-Funel, 2001), histidine decarboxylase in *S. thermophilus* (Tabanelli et al., 2012) and Gram-negative bacteria such *as Photobacterium phosphoreum, Photobacterium damselae, M. morganii*, and *R. planticola* (Kanki et al., 2007).

In general, the data reported indicate a great variability in the response of the cell decarboxylase systems to the environmental factors, which reflects differences among species and genus metabolic pathways, experimental conditions, type of matrix (food) considered, but also it is the results of the great heterogeneity characterizing decarboxylase activity, even within strains of the same species.

Even if the environmental factors significantly affect the rate and the entity of BA accumulation, in fermented foods their modulation is often limited by the conditions, which allow the fermentation and ripening processes (in turn linked to the “traditional” features of the products) and by health trends, as in the case of the reduction of NaCl content.

### Temperature

Temperatures close to the optimum growth values, promoting cell metabolism and proliferation, generally favor the production of BAs, which is often related to the number of cells present in the system. However, the presence of high number of decarboxylating cells is not a sufficient condition to explain the final BA amount (Marcobal et al., 2006b, 2012).

Studies carried out in a model system using a *E. faecalis* EF37, demonstrated that the increase of temperature from 16 to 44°C coincided with a faster growth and with a more rapid and intense accumulation of tyramine (Gardini et al., 2001). More recently, Bargossi et al. (2015a) studied the effect of temperature on the activity of a pure commercial tyrosine decarboxylase extracted from *E. faecalis* and found the highest decarboxylation efficiency at a temperature comprised between 30 and 37°C. By contrast, Zhang and Ni (2014) found that a tyrosine decarboxylase from *L. brevis* had its optimum temperature at 50°C, but it was rapidly inactivated at higher temperature. However, the activity of the enzyme at the optimum temperature was rapidly decreased during the permanence at 50°C for an hour.

Cells of *E. faecalis* and *E. faecium* strains suspended in buffered systems containing tyrosine and incubated at different temperatures confirmed the maximum decarboxylase activity at 37°C after 2 h of incubation. However, after 24 h the maximum tyramine content was surprisingly found in the sample incubated at the less favorable temperature (20°C; Bargossi et al., 2015a).

Using an experimental design in which several parameters were taken into consideration, Marcobal et al. (2006b) found that the optimum temperature for tyramine production under aerobic condition by *E. faecium* and *L. brevis* was 32°C. By contrast, under anaerobic conditions, the maximum tyramine concentration was obtained at 22.0–24.5°C.

The histidine decarboxylase of a cell free extract of a *S. thermophilus* strain had its maximum activity at 50°C. It decreased rapidly at higher temperature (60°C), while it maintained a detectable activity at 5°C (Tabanelli et al., 2012). By contrast, active cells of the same strain produced more rapidly histamine at 40°C, while the BA accumulation was limited or negligible at 25 and 20°C within the incubation period considered. In addition, an histamine producing strain (*S. thermophilus*) incubated at low-temperature (4°C) in milk produced less histamine than did the same strain kept at 42°C. This reduction was attributed to a lower activity of the histidine decarboxylase enzyme rather than to a reduction in gene expression or the presence of a lower cell number (Calles-Enríquez et al., 2010).

Regarding Gram-negative bacteria, Morii and Kasama (2004) studied in *P. phosphoreum* cell free extract the activity of two different histidine decarboxylases and found that the inducible enzyme had its maximum activity at 30°C while the constitutive one at 40°C. The optimum growth temperature of the strain was 25°C while the specific activity of histidine decarboxylase of cell free extracts was extremely high in the cells grown at low temperature (7°C). The histamine producing potential of the Gram-negative bacterium *Mycobacterium psychrotolerans* was studied in relation to the temperature in the range 0–20°C. Increasing temperature enhanced the rate of accumulation but also the final tyramine accumulation (Emborg and Dalgaard, 2008). The optimum temperature for histidine decarboxylase activity of bacteria belonging to different species (*M. morganii, R. planticola, P. phosphoreum*, and *P. damselae*) ranged between 30 and 40°C. It was still active at 5°C but not at 60°C (Kanki et al., 2007).

Scarce reports are available concerning the relation between other BAs (putrescine, cadaverine, and tryptamine) and temperature. Generally, the accumulation of BAs, among which cadaverine and putrescine increased with temperature (Wunderlichová et al., 2014); nevertheless, prolonged storage at low temperatures can result in accumulation of putrescine explained by the metabolism of psychrotrophic pseudomonads (Paulsen and Bauer, 1997).

Bubelová et al. (2015) studied the production of putrescine and cadaverine in relation to temperature in *Serratia marcescens*. They found that the maximum amount of these two BAs was reached at 20–30°C. If the production was compared to the biomass (“yield factor”), the decarboxylase activity of the single cells was maximum at 10°C.

In general, the ability to produce BAs is limited by the decreasing of the temperature. This implies that the control of the cold chain during storage and commercialization is a main tool to avoid the accumulation of undesired products after manufacturing, especially in not fermented foods, such as fishery products (Knope et al., 2014). Several authors stressed the crucial effect of the storage temperature on the histamine formation (and other BAs) in fish such as tuna (Veciana-Nogués et al., 2004; Emborg and Dalgaard, 2006; Kanki et al., 2007) and anchovies (Veciana-Nogués et al., 1997; Visciano et al., 2007).

Regarding fermented foods, the temperature of fermentation and during ripening has to allow the microbiological activity of the desired microbiota and the range within they can be modulated is rather strict, defined by the protocols for the production of the different fermented food typologies. The temperature applied during the first 3 days of fermentation of dry sausages influenced the BA accumulation (tyramine, 2-phenylethylamine, cadaverine, and putrescine) during all the ripening period (1 month) with increasing values in the presence of higher temperature, which was between 15 and 25°C (Gardini et al., 2008; Bover-Cid et al., 2009). Higher fermentation temperatures (and higher relative humidity) favored tyramine and 2-phenylethylamine accumulation in the Spanish fermented sausages Fuet and Llonganissa inoculated with *L. curvatus* (Latorre-Moratalla et al., 2012). By contrast, no differences were found in Turkish sausages ripened at 22 or 26°C (Gücükoğlu and Küplülu, 2010).

Ruiz-Capillas et al. (2007) found that tyramine was accumul-ated in higher amounts in pressurized sliced cooked ham packaged under vacuum when the storage temperature was higher. Also fermented sausages stored after production under room temperature were characterized by higher BA content than refrigerated products (Komprda et al., 2001).

A higher tyramine content was found in salted duck inoculated with *E. faecalis* stored at 20°C compared with the samples incubated at 4°C; however, no differences were found in the tyrosine decarboxylase gene expression indicating an univocal effect of temperature on enzymatic activity (Liu et al., 2014). Also in green fermented olives, the adoption of a low fermentation temperature reduced the accumulation of cadaverine and tyramine together with the formation of “zapatera” defect (García García et al., 2004).

The application of thermal treatments (when possible) to raw material such as milk before fermentation (pasteurization) can contribute to the elimination of the wild decarboxylating microbiota. The Gram-negative BA producers (enterobacteria and pseudomonads) are rapidly inactivated by temperatures higher than 60°C. LAB are more resistant and require more drastic thermal treatments. For this reason, usually cheeses from pasteurized milk are characterized by lower BA content (Schneller et al., 1997; Novella-Rodríguez et al., 2003; Novella-Rodríguez et al., 2004; Marino et al., 2008). However, Ladero et al. (2011) observed the survival in skim milk of a tyraminogenic strain of *L. curvatus* after a treatment at 78°C. Independently of the cell viability, the information concerning the thermal stability of decarboxylase is scarce. The histamine decarboxylase produced by *S. thermophilus* maintained a residual activity after treatments at 70 and 75° for 10 min (Tabanelli et al., 2012). In any case, the pasteurization of milk does not avoid the presence of a ripening microbiota, which can contribute to BA accumulation. Pinho et al. (2001) found a relevant increase in BA content of a Portuguese cheese (Azeitao) when the storage temperature increased from 4 to 25°C, related to the higher protease activity. In addition, it has been observed that the BAs content was higher in Dutch type cheese when the temperature of ripening and storage increased (Buňková et al., 2010; Pachlová et al., 2012).

### Salt Concentration

In general, increasing salt concentrations contribute to the reduction of BA accumulation in foods, mainly reducing the metabolic activities of decarboxylating microorganisms. In particular, Gram-negative bacteria are more inhibited by increasing salt concentrations than Gram-positive microbiota. However, the health trend to reduce NaCl concentration is in contrast with this possible tool to reduce BA accumulation in foods.

The pure tyrosine decarboxylase tested by Bargossi et al. (2015a) demonstrated a limited loss of its relative activity in the presence of increasing amount of NaCl up to 10%. Only the addition of 15% of salt determined a more marked reduction of the decarboxylation potential, which remained, however, higher than 50% of the activity recorded in the absence of salt added.

In an *E. faecalis* strain (EF37) grown on a synthetic medium, the ability to accumulate tyramine and phenylethylamine was inversely related to the NaCl concentration, in a range comprised between 2 and 6%, while the proteolysis showed an optimum between 2 and 3% (Gardini et al., 2001). In fermented sausages inoculated with the same tyraminogenic *E. faecalis* strain, increasing amounts of salt reduced the concentration of tyramine, 2-phenylethylamine produced by enterococci, but also limited cadaverine and putrescine production by enterobacteria (Gardini et al., 2008; Bover-Cid et al., 2009).

The tyramine production of different strains of *E. faecalis* and *E. faecium* was studied in buffered systems containing tyrosine; the results indicated that *E. faecalis* partially reduced its tyraminogenic potential of cells passing from 0 to 5% of NaCl but the decarboxylation activity did not change significantly increasing NaCl concentration up to 15%. On the other hand, the same enzymatic activity in cells of *E. faecium* remained quite constant independently of the NaCl concentration (Bargossi et al., 2015a). Strains of *Lactococcus lactis* ssp. *lactis* and *L. lactis* ssp. *cremori*s reached their maximum tyramine production in synthetic medium only in the presence of the higher salt concentration used in the trials (2%; Buňková et al., 2011); in addition the same condition allowed the maximum tyramine production rate as well as the lowest time for the production of detectable amounts of the BA.

A different role of salt on the histidine decarboxylase activity in a strain of *S. thermophilus* was highlighted in viable cells and in cell free extract. While the production of histamine was almost completely prevented in living cells by a salt concentration of 2.5% (by limiting or inhibiting the growth potential of *S. thermophilus*), the activity of the decarboxylase in cell free extract was unaffected up to 5% NaCl and then slowly decreasing, maintaining an activity, even if reduced, at 20–30% NaCl (Tabanelli et al., 2012). The presence of NaCl led to an up-regulation of histidine decarboxylase gene in the same strain grown on skim milk, suggesting a potential role of this enzyme also in osmoprotection mechanisms (Rossi et al., 2011). This confirmed that the activation of decarboxylase systems is a part of complex metabolic responses in the presence of different stress conditions (Pessione et al., 2009).

A halophilic LAB strain of *Tetragenococcus muriaticus* isolated from fish sauce produced histamine during the late exponential growth phase, reached a maximum production of this BA at 5–7% of NaCl, and was able to maintain a histidine decarboxylase activity also in the presence of 20% of salt (Kimura et al., 2001). On the other hand, the histamine decarboxylase activity of two strains of *P. phosphoreum* decreased rapidly with the increase of salt (2–5% of the optimum activity in the presence of 10% of NaCl), while more resistant (40–50% of the optimum activity) resulted the activity of the same enzyme in strains of *R. planticola, P. damselae*, and *M. morganii* (Kanki et al., 2007). Using a strain of *P. phosphoreum*, Morii and Kasama (2004) found that histidine decarboxylase activity in cell free extract was higher at level of 5% NaCl, while the cells were not able to multiply (and produce BA) under this salt concentration. Similar effects were described also for Bifidobacteria (Lorencová et al., 2014). The hypothesis of this possible enhancing effect on the BA production of NaCl has been found by some authors in the essential role of Na^+^ ion in the sodium/proton antiport system through which H^+^ ions are removed from the cell (Pereira et al., 2009; Buňková et al., 2011; Lorencová et al., 2014; Bubelová et al., 2015).

*Serratia marcescens* produced putrescine and cadaverine with more efficiency in the presence of 1–3% NaCl (3–5 in the yield factor was applied; Bubelová et al., 2015). Cells of *M. psychrotolerans* produced more histamine when grown in the presence of 4% NaCl compared with lower salt concentration (Emborg and Dalgaard, 2008) even if this salt concentration slowed the growth of the strain. In other words, stressed cells seem to activate the decarboxylating pathways in the framework of more complex response systems. This make the potential of BA production by each single cell more efficient. Also *E. aerogenes* produced the maximum amounts of cadaverine, putrescine, and histamine in the presence of the 3% of NaCl (Greif et al., 2006).

In fermented sausages, BAs are accumulated during ripening. However, the rate of accumulation decreases with the decrease of a_w_ due to the water losses. Products packaged under modified atmosphere packaging (MAP), in which the weight losses were inhibited, continued to accumulate BAs when the packaging was carried out at high a_w_ (0.92 and more; González-Tenorio et al., 2013; Tabanelli et al., 2013). The Greek cheese Feta, characterized by a high salt content, with a ripening carried out in brine and with a low pH, was characterized by a noteworthy amine concentration (about 200 mg/kg of tyramine, 90 mg/kg of histamine, and 200 mg/kg of putrescine; Valsamaki et al., 2000).

### pH

Since the decarboxylation is a mechanism of cells to counteract acidic stress, it is clear that several studies were focused on the study of the relationships between pH and BA accumulation. Also in this case, the effect of pH is different if the focus is directed toward the activity of the pure enzyme or the decarboxylase activity of the living cells. In any case, it has been extensively demonstrated that the transcription of genes of many decarboxylase clusters are induced by low pH and improves the fitness of cells subjected to acidic stress (Pereira et al., 2009; Pessione et al., 2009; Marcobal et al., 2012; Romano et al., 2012, 2014; Perez et al., 2015)

A commercial pure tyrosine decarboxylase had its maximum activity in buffered systems at pH between 5 and 6 (Bargossi et al., 2015a), while at pH 4 the same activity was extremely weak. The tyrosine decarboxylase obtained from a strain of *L. brevis* had its maximum relative activity at pH 5, while it maintained the higher stability at pH 7.4, e.g., 92% activity retained after 7 days of incubation (Zhang and Ni, 2014).

By contrast, a strain of *E. faecalis* under the same conditions showed the maximum tyramine accumulation at pH 4 after 2 h of incubation and pH between 4 and 5 after 24 h. In the same system, a strain of *E. faecium* did not show relevant differences in its decarboxylase activity at pH between 4 and 6. No pH differences in relation to the production of tyramine were observed in whole cells or cell free extract of *L. brevis*, with optimum activity at pH 5 (in the range 2–9); however, the cell-free extract had a higher activity compared with the whole cells (Moreno-Arribas and Lonvaud-Funel, 2001). Two tyrosine decarboxylases from *E. faecalis* and *E. faecium* (heterologously expressed in *E. coli*) had their optimum pH for the activity at 5.5 and 6, respectively (Liu et al., 2014).

The enhancing effects of lower pH on tyramine production (as responses to acidic stress) was observed also in *Enterococcus durans* (Fernández et al., 2007), *E. faecium* (Marcobal et al., 2006a; Pereira et al., 2009). Similar effects were observed for the histidine decarboxylase in *L. lactis* (Trip et al., 2012) and *L. brevis* (Marcobal et al., 2006b).

The histidine decarboxylase of *S. thermophilus* has its optimum pH at pH 4.5, measured in cell free extract, while histamine accumulation by viable cell cultures was very low at the same pH, due to the negative effect of acidity on the overall metabolism of the strain (Tabanelli et al., 2012).

Regarding Gram-negative bacteria, the pure histidine decarboxylase from *P. phosphoreum* had its higher activity at pH 7; this value decreased at 6.0 for *P. damselae* and 6.5 for *M. morganii* and *R. planticola* (Kanki et al., 2007). Morii and Kasama (2004) found that an optimal histidine decarboxylase activity was slightly lower (pH 6) in *P. phosphoreum*, while BA production by cells of *Enterobacter cloacae* and *E. aerogenes* was higher at pH 6 (Greif et al., 2006).

Cid et al. (2008) suggested that in fermented sausages, the accumulation of tyramine by LAB (*L. curvatus*) started immediately at the end of fermentation, when pH of the sausages has already reached its minimum value. Other BAs, produced by the same LAB, were produced more gradually only at a later stage of ripening.

## Technological Factors for Controlling Biogenic Amine Accumulation

### Use of Starter Culture

The addition of selected starter cultures is one of the main tools able to counteract BA accumulation in fermented foods. In first instance, the microorganisms used have to be characterized by the absence of any decarboxylating activity. Then, they had to be active in inhibiting the growth performances and the aminobiogenetic potential of wild decarboxylating bacteria. In addition, aspects such as the production of antimicrobial compounds like bacteriocins, as well as the ability to degrade BAs have to be considered (Ayhan et al., 1999; Bover-Cid et al., 2000). The possibility of some microorganisms to metabolize BA will be specifically discussed in Section “Microorganisms Able to Metabolize Biogenic Amine.” The use of LAB cultures producing bacteriocins can have an important potential in limiting BA accumulation, even if further researches are needed to clarify this potential. Recently it has been demonstrated that using bacteriocinogenic strains of *L. lactis* it is possible to limit BA production by *S. thermophilus* and *E. faecalis* (Tabanelli et al., 2014).

The use of starter cultures has a consolidated application in cheese and the selection of not decarboxylative cultures for dairy products is well established since long time (Cogan et al., 2007). The use of selected starter cultures aimed to limit BA accumulation in dairy products has been recently reviewed by Linares et al. (2012). The use of autochthonous starter has been tested successfully in ewe’s milk cheeses by Renes et al. (2014) who found a significantly lower BA content when *L. lactis* starter cultures were used. These results confirmed the observation of Novella-Rodríguez et al. (2002a) regarding goat cheeses. Also in Manchego cheese, the use of autochthonous starter cultures of *L. paracasei* decreased the accumulation of BA, even if compared with commercial starter cultures (Poveda et al., 2015).

European Food Safety Agency (EFSA) recommends that so-called autochthonous strains (i.e., selected strains originating from each specific fermented product) with suitable technological profiles and reduced tendencies to produce BA should be selected as starter cultures (EFSA, 2011). Recently, it was suggested to use autochthonous starter cultures for fermentation of artisanal sausages (Talon et al., 2007; Casquete et al., 2011). Latorre-Moratalla et al. (2012) have extensively reviewed many of these aspects relevant in fermented sausage production. The addition of pure or mixed selected starter cultures can decrease BAs accumulation in sausages. Mixed starters were reported to perform better than single starters to control the growth of different bacterial groups (Latorre-Moratalla et al., 2010b; Naila et al., 2010). In fermented sausages, autochthonous starter cultures determined BA reductions higher (Latorre-Moratalla et al., 2010a; Casquete et al., 2011; Renes et al., 2014) than commercial mixed starter cultures (Ayhan et al., 1999; Gücükoğlu and Küplülu, 2010). In fact, Latorre-Moratalla et al. (2012) reported that mixed starter cultures of amine negative strains of LAB and coagulase-negative staphylococci, well adapted to the meat fermentation environment, were the best choice to reduce BA content in sausages. In order to avoid formation of high level of BAs during fermentation of sausages, the use of raw materials with low microbial counts is recommended. The main level of BA production was during the first 3 days, when a sharp pH decrease and the development of LAB occurred during the fermentation process of dry sausages (Bover-Cid et al., 1999). LAB used as starter cultures can induce rapid acidification, thus inhibiting the growth of decarboxylating microorganisms, resulting in decreasing formation of BAs (Zhang et al., 2013). However, it is well known that the use of selected starter cultures alone cannot assure the reduction or inhibition of BA production (Parente et al., 2001).

The production of BA in wine is mainly associated with the activity of LAB (Lonvaud-Funel, 2001). For this reason, particular attention has been posed on the selection of starter cultures used for malolactic fermentation. Strains of *O. oeni*, the main responsible for the conversion of malic acid into lactic acid in wine, can produce histamine (Lonvaud-Funel and Joyeux, 1994; Guerrini et al., 2002) and tyramine (Gardini et al., 2005) and selected strains without this potential should be used for the conduction of this process in winemaking (Moreno-Arribas et al., 2003).

Also in the production of sauerkraut, the use of starter cultures can contribute to the reduction of BA content (Kalač et al., 2000; Rabie et al., 2011a).

### Technological Additives

The use of some additives is widespread to improve the appearance and the quality of the final product such as development of the typical color of the cured meat, inhibition of mold growth and decrease of toxic compounds in the product. Thus, the effects of these additives on BA generation are important.

The scarcity of sugars, and in general poor nutritional environments, has been often associated to higher BA accumulation, being decarboxylative pathways secondary transport system providing metabolic energy (Konings, 2006). For this reason, the *in vitro* production of BAs has been tested by several authors in relation to sugar supply. Buňková et al. (2012) found the maximum tyramine accumulation by *E. durans* in the presence of the higher lactose concentration (5%). A less clear effect of this sugar was observed for *L. lactis*: increasing lactose concentration did not result in higher tyramine concentration and this was attributed to the excess of metabolic energy obtained by primary fermentation (Buňková et al., 2011). Higher amount of tyrosine were produced by *S. thermophilus* in the presence of limiting amounts of lactose (0.1%; La Gioia et al., 2011). Also Landete et al. (2008b) observed that increasing concentration of fructose and glucose progressively inhibited the histamine accumulation of enological LAB belonging to the species *L. hilgardii, P. parvulus*, and *O. oeni*.

In industrial formulations, especially in fermented sausages, sugars (mainly glucose, sucrose, and lactose) are added in order to improve the LAB fermentation process. González-Fernández et al. (2003) investigated the influence of three decarboxylase negative starter cultures in relation to different composition and concentration of sugars, on the presence of BAs in *chorizo*, a typical Spanish dry fermented sausage. The highest concentrations of BAs were found at the end of the ripening process in the control sausage with no starter culture irrespective of the use of different sugar concentrations. However, when a starter culture and sugar concentrations equal to 0.5% or 1% were used, the presence of BAs in the sausage decreased considerably in comparison with control and low sugar concentration sausages. The production of high amounts of putrescine (223–252 mg/kg) and tyramine (64–102 mg/kg) was observed when the concentration of sugar in the sausage was only 0.1%, even in sausages with a starter culture added. Bover-Cid et al. (2001a) also found contents of tyramine and cadaverine significantly higher in sausages without sugar in their formulation. They concluded that sugar omission is not recommended since it might increase BA accumulation during the manufacture and storage of slightly fermented sausages. The amount of sugar added can be a key factor in determining the equilibrium among the microbial communities during the fermentation step of sausages favoring the accumulation of different BAs. The modulation of sugar addition between 0 and 1.4% determined the maximum tyramine content at intermediate level, in correspondence with the maximum LAB and enterococci growth. By contrast, at the extreme levels (0 and 1.4%), the accumulation of putrescine and cadaverine was higher, associated with the best performance of enterobacteria (Bover-Cid et al., 2009). High accumulation of cadaverine and putrescine were observed also in chorizo in the presence of low sugar addition (0.1%) even in the presence of starter cultures added (González-Fernández et al., 2003).

The use of essential oils as aroma and flavor ingredients has increased recently because of the growing consumer demand for natural products as natural food preservatives and the replacement of synthetic additives in the food industry. Wendakoon and Sakaguchi (1993) reported that antibacterial activity of essential oil such as eugenol present in clove might delay the BA formation of *E. aerogenes* in mackerel muscle extract. No amines were observed in blue fish burgers treated with thymol, lemon extract, and grapefruit seed extract combination with MAP (Del Nobile et al., 2009). Özogul et al. (2015) investigated the impact of carvacrol at different level (0.1, 0.5 and 1 ml/100 ml) on BAs production by foodborne pathogens including, *Staphylococcus aureus, E. coli, Klebsiella pneumoniae, E. faecalis, Pseudomonas aeruginosa, Listeria monocytogenes, A. hydrophila*, and *Salmonella* Paratyphi A in histidine decarboxylase broth. The results of this study showed that all bacteria tested were able to decarboxylate more than one amino acid and that carvacrol was able to reduce BAs formation depending on its concentration and bacterial species. Cai et al. (2015) reported low BAs content, especially histamine, putrescine, cadaverine in red drum (*Sciaenops ocellatus*) filets treated with 4 ml/l clove, cumin, and spearmint oils. In addition, Bozkurt (2006) reported that the levels of histamine, putrescine, and tyramine in *sucuk* (Turkish dry-fermented sausage) were lower in sausages with green tea extract than control. In Gouda cheese, the addition to curd of *Zataria multiflora* essential oil, whose major constituent was carvacrol (71.1%) determined a significant reduction of tyramine and histamine in the final product (Gorji et al., 2014).

In this perspective, spices can affect BA contents in food. Spices are often defined as aromatic, dried plant substances used in foods for flavoring and coloring. Komprda et al. (2004) reported that high content of red pepper, together with starter cultures, contributed to the lower BA content in dry fermented sausages. Red pepper contains capsaicin, known to prevent the growth of some bacteria (Hirasa and Takemasa, 1998). The effects of a variety of spices including ginger, garlic, green onion, red pepper, clove, and cinnamon were investigated to reduce BA contents in *myeolchi-jeot*, Korean salted and fermented anchovy (Mah et al., 2009). The greatest inhibitory effect on BA production was found in the culture treated by garlic extract while the other spice extracts showed minor effect in reducing BA contents. In particular, ginger extract reduced putrescine contents while red pepper extract decreased cadaverine accumulation. Since garlic contains allicin, antimicrobial component, its efficacy is probably related to the presence of this molecule. Other studies (Shakila et al., 1996) reported that clove and cinnamon reduced histamine production of *M. morganii* by 95%.

### Effects of Packaging on Biogenic Amine Formation

There is evidence that oxygen can affect the BA production. For example, it has been demonstrated that *E. durans* (Buňková et al., 2012) and *L. lactis* (Buňková et al., 2011) tyramine accumulation is favored by anaerobic conditions. A similar trend was reported also by Cid et al. (2008) using a strain of *L. curvatus*. However, the main technologies for food preservation based on atmosphere modification are focused on oxygen exclusion. Nevertheless, in such strategy, the principal aim, in relation to BA presence, is not the inactivation of decarboxylase activity but the inhibition of microbial population with decarboxylating properties.

In this perspective, the atmosphere used for packaging can affect the qualitative and quantitative formation of BAs. MAP and vacuum packaging (VP) play an important role in the selection of spoilage microorganisms and, particularly, on decarboxylating bacteria (Curiel et al., 2011).

CO_2_ is the main gas used as bacteriostatic agent. Different concentrations of this gas in MAP have been applied to prolong shelf-life of foods by inhibiting microbial growth of Enterobacteria and H_2_S-producers bacteria (Lopez-Caballero et al., 2002), histamine forming bacteria (Özogul and Özogul, 2006), *Pseudomonas* spp. (Li et al., 2014), *Lactobacillus sakei* (Devlieghere et al., 1998) and psychrophilic microorganisms (Arashisar et al., 2004). The CO_2_ concentration (>60%) in the MAP caused significant reductions in the contents of total BAs in barramundi filets (Yassoralipour et al., 2012) and sardine (Özogul and Özogul, 2006). Rodrigues et al. (2016) also indicated that lower production of putrescine and cadaverine was observed in MAP (80% CO_2_/20% N_2_) and VP samples of rainbow trout. Yew et al. (2014) investigated the major BA profile in Indian mackerel packed in different carbon dioxide compositions (30, 60, 80, and 100% CO_2_) with content 5% O_2_ and corresponding N_2_ level. Each amine responded differently to different CO_2_ levels. Histamine concentration was reduced by 6.4, 8.5, 70.3, 78.8, and 90.2% in fish packed under VP, 30, 60, 80, and 100% CO_2_, respectively. In particular, histamine and tyramine increased rapidly in fish packed under VP and 30% CO_2_. This was attributed to the presence of histidine and tyrosine decarboxylase bacteria that cannot grow in the presence of increasing CO_2_ concentration (Barakat et al., 2000).

LAB, due to their capacity to grow under high concentrations of CO_2_, constitute a substantial part of the natural microbiota of MAP meats (Chouliara et al., 2007). Curiel et al. (2011) demonstrated that under vacuum conditions or MAP (20% CO_2_ and 80% N_2_) the enterobacteria producing putrescine, cadaverine, and agmatine were inhibited. In addition, the packaging under vacuum did not reduce significantly the tyraminogenic potential of strains of *Carnobacterium divergens*.

Zhang et al. (2015) evaluated the effect of air and MAP on the shelf life of chilled chicken. The putrescine and cadaverine content of gas mixture-packaged samples was significantly lower than that of the air-packaged samples during the storage period. The production of these BAs was slowed as the CO_2_ content increased, indicating that the increasing concentration of CO_2_ inhibited the growth of putrescine and cadaverine producing bacteria. The results are in agreement with those reported by Rodriguez et al. (2015) who investigated the effect of CO_2_ concentration on the formation of BAs in shredded cooked chicken breast filet packed in modified atmosphere. They reported that the putrescine and cadaverine are affected by storage time and CO_2_ concentration. Putrescine-producing bacteria are more CO_2_-resistant. The correlation between BA formation and bacterial growth showed that putrescine and cadaverine concentrations could be used as quality indicators as their formation is related to total viable count.

Nowadays the most widely used active packaging technology for food is oxygen scavengers which eliminate oxygen in the packaging and in the product or permeating through the packaging material during storage (Alvarez, 2000). Mohan et al. (2009) investigated the effect of O_2_ scavenger on the formation of BAs during chilled storage of seer fish (*Scomberomorus commerson*) and indicated that the use of O_2_ scavenger with the proper maintenance of chilled storage temperature helped in reducing the formation of BAs and also reduced the risk of *Clostridium botulinum* toxin. Similar results using commercial O_2_ scavengers were reported by Mohan et al. (2008) for catfish (*Pangasius sutchi*) steaks and Goncalves et al. (2004) for gilthead sea bream (*Sparus aurata*).

Naila et al. (2010) indicated that active packaging, VP and MAP inhibit formation of BAs more effectively than air packaging, through inhibition of BA forming bacteria or enzyme activity.

### Other Non-thermal Treatments

Food irradiation can be used to increase the safety and shelf life of foods by reducing microbial growth. Gamma-irradiation has been reported to reduce BAs contents in food due to the delay in the initial growth of adventitious microorganisms (Kim et al., 2003, 2004). Low levels of BAs by gamma irradiation in pepperoni sausage during storage were observed (Kim et al., 2005a). Gamma irradiation at 5, 10, or 15 kGy reduced putrescine, cadaverine, agmatine, histamine, tryptamine, spermine, and spermidine during fermentation of low-salt fermented soy paste (Kim et al., 2005b). Rabie et al. (2010) investigated the effects of different doses (2, 4, and 6 kGy) of gamma irradiation on BA formation in Egyptian fermented sausages. Histamine was detected in irradiated samples, immediately after irradiation, but not afterward and low levels of all BAs were observed during storage period especially in products treated at 6 kGy. Similar results were obtained for Blue cheese (Rabie et al., 2011b). Chub mackerel (*Scomber japonicus*) in chilled storage, after irradiation followed by vacuum packing slowed down the formation of BAs (Mbarki et al., 2009). Özogul and Ozden (2013) reported that radiation levels of (2.5 and 5 kGy) had similar effects on reducing the BA content except for agmatine and tryptamine. Although radiation caused an increase in spermine, agmatine, and tryptamine content in sea bream muscle, the putrescine and cadaverine contents in this product significantly decreased following the radiation process.

Pulsed electric fields (PEF) is a non-thermal method of food preservation that uses short pulses of electricity for microbial inactivation and causes minimal detrimental effect on food quality attributes (Quass, 1997). Previous studies have proven that PEF treatment can be useful in the sterilization of fruit juice under various conditions (Gurtler et al., 2010; Guo et al., 2014). They observed a clear microbial inactivation, depending on the juice, microorganisms, treatment conditions, and equipment. In addition, increasing the electric field strength (>600 kV/m) improved the inhibition of microorganism growth in tilapia during storage (Ko et al., 2016).

### Microorganisms Able to Metabolize Biogenic Amine

Many microorganisms can produce amino oxidases, which are the enzyme responsible for BA detoxification. These enzymes metabolize BAs firstly by deamination, with the production of NH_3_ and H_2_O_2_ in the presence of oxygen. The aldehydes formed is further reduced to the corresponding acids which can be then transferred to the central metabolism of the cells (Cooper, 1997). This metabolic pathway can be used as a source of NH_3_ in nitrogen poor media. These enzymatic activities have been evidenced *in vitro* in several microorganisms. Leuschner et al. (1998) found these abilities widespread among *Kocuria* (former *Micrococcus*) *varians.* In addition, they isolated amino oxidase positive *Brevibacterium linens* strains, which were used for the production of a surface ripened Munster cheese, causing the reduction of BA (Leuschner and Hammes, 1998).

Amino oxidase activity was found also in *Bacillus amyloli-quefaciens* and *Bacillus subtilis* (Zaman et al., 2010, 2011). Among LAB, many strains of *Lactobacillus, Pediococcus*, and *Oenococcus* showed these ability in culture media (García-Ruiz et al., 2011). Similar activities were observed *in vitro* also in *Lactobacillus casei, Lactobacillus plantarum* (Fadda et al., 2001; Herrero-Fresno et al., 2012), and in *L. sakei* in ensiled fish slurry (Dapkevicius et al., 2000). Herrero-Fresno et al. (2012) demonstrated also that the use of two strains of *L. casei* with high degradation rates for histamine and tyramine could reduce the accumulation of these BAs in Cabrales-like mini-cheeses.

Also staphylococci were able to deaminate tyramine and histamine *in vitro* (Martuscelli et al., 2000; Zaman et al., 2011). However, the activity of selected staphylococci in real food systems was limited by the presence of other nitrogen sources easily available for these bacteria, such in the case of dry fermented sausages (Gardini et al., 2002). In addition, in real complex food systems, the BA reduction by an amino oxidase positive *L. casei* strain cannot be clearly ascribed to an effective BA deamination or to a specific antagonism of the strains toward decarboxylating microorganism (Nishino et al., 2007). More interesting results were obtained in a nitrogen poor matrix such as wine, in which the use of strains of *L. casei* and *Pediococcus* spp. (García-Ruiz et al., 2011), as well as *L. plantarum* (Capozzi et al., 2012), determined a BA deamination.

Finally, some authors suggested the possibility to use purified amino oxidase in foods with negligible results (Hobson and Anderson, 1985; Dapkevicius et al., 2000). More interesting reductions were observed in wine using fungal amino oxidases (Cueva et al., 2012).

### Effects of Pressure Treatments on BA Formation

Among the alternative processes to thermal treatment for food preservation, the field of high pressure processing, better known as high hydrostatic pressure (HHP) or high pressure homogenization (HPH), is one of the most scientifically explored. Given the antimicrobial effects of HHP, this technology can modify the microbiota of treated foods both quantitatively and qualitatively, affecting also the food matrix characteristics (Georget et al., 2015). Novella-Rodríguez et al. (2002b) studied the differences in BA formation in goat cheeses by using pasteurized and pressurized milk and found no significant effects between the two trials on tyramine, histamine, and putrescine accumulations. The treatment at 400 MPa or 600 MPa of 21- and 35-day ripened cheeses determined a significant increase of both aminopeptidase activity and free amino acid concentration. By contrast, the total BA concentration was higher in the not treated cheeses, especially in samples treated at 600 MPa (50% and more of BA reduction; Calzada et al., 2013). In other words, HHP treatment reduced the population of potentially decarboxylating microorganisms, limiting BA accumulation also in the presence of a higher concentration of precursors. Novella-Rodríguez et al. (2002a) used HHP to accelerate cheese ripening, founding no differences in tyramine content between treated and not-treated goat cheese samples when 400 MPa were applied; however, in the same work, a prolonged treatment at 50 MPa increased the tyramine content.

Ruiz-Capillas et al. (2007) studied the BA formation in sliced dry-cured “chorizo” sausage, HHP treated at 350 MPa for 15 min, during chilled storage at 2°C and found a significant reduction of tyramine, putrescine, and cadaverine concentrations. The reason for this decrease was found in the higher susceptibility of decarboxylating microorganisms. However, during the storage, BAs continued to increase, probably due to the residual decarboxylases, whose activity was independent on the cell viability. Ruiz-Capillas et al. (2007) described a positive effect of HHP treatment of 400 MPa for 10 min on tyramine accumulation in vacuum packaged cooked sliced ham, with an increase of the shelf-life by at least 35 days. Similar results were observed by treating at 500 MPa/10 min Hungarian dry fermented sausages cut in 5 cm long pieces under vacuum (Simon-Sarkadi et al., 2012). Under these conditions, BA content was reduced during the storage.

In addition to HHP, also HPH can be a strategy to control the presence of decarboxylating microorganisms in raw materials such as the milk used for cheese making. Lanciotti et al. (2007) demonstrated that a HPH milk treatment at 100 MPa can significantly reduce the BAs accumulation compared with the thermal treated milk, due to a deep modification of the microbiota during ripening.

### Antimicrobial Substances

The use of substance with antimicrobial properties can modify the BA profile of foods interfering with the equilibrium among microbial population rather than affecting directly the decarboxylase efficiency. Nitrate and nitrite salts are commonly used in fermented sausages for different purposes. In fact, they influence the color, the flavor and the oxidation of cured meat. In addition, they are used for controlling hazardous bacteria, such as clostridia. Nevertheless, their action can also interfere with BA accumulation. The addition of increasing concentration of nitrite (up to 150 mg/kg) reduced tyramine and cadaverine accumulation in Sucuk, a Turkish fermented sausage (Gençcelep et al., 2008). Opposite results were obtained in a Spanish not fermented cured meat (Lacon) in which the addition of nitrate and nitrite significantly increased the BA content (Lorenzo et al., 2007). This trend was explained by the fact that the addition of nitrite favored the selection of a superficial microbiota mainly constituted by LAB with relevant decarboxylative activity. The addition of nitrite reduced also the BA content in fresh beef, pork, and poultry meats stored at 4°C (Jastrzkebska et al., 2016). These latter authors tested, under the same condition, also a weak acid (sorbic acid) whose effectiveness was rather limited. The use of two weak acids (sorbate and benzoate), in combination with clove, inhibited the decarboxylative action of an aminobiogenetic *E. aerogenes* strain isolated from fish (Wendakoon and Sakaguchi, 1993). Benzoate and sorbate were also used to limit BA production during the storage of two different type of fishery products (cod roe and pearl mullet filets, respectively; Lapa-Guimarães et al., 2011; Gençcelep et al., 2014).

Also sulfur containing antimicrobials were used to control BA accumulation in foods. Bover-Cid et al. (2001b) added sodium sulfide (maximum concentration 1000 mg/kg) to ripened sausages and observed a contradictory effect on BA accumulation. In fact, while the cadaverine content was inhibited by the addition of sulfide, tyramine, and putrescine accumulation was strongly enhanced. Jastrzkebska et al. (2016) observed a contribution of sodium metabisulphite to the reduction of BA content in fresh meats. However, the most relevant matrices in which sulfur compounds play a key role in the reduction of BAs are the alcoholic beverages. In particular, in wine it is known the potential aminobiogenetic potential of the LAB responsible for malolactic fermentation (Lonvaud-Funel, 2001; Landete et al., 2005). Some authors demonstrated that the addition of SO_2_ when malate was completely converted into lactate prevented amine formation in subsequent stages (Vidal-Carou et al., 1990; Marcobal et al., 2006c). A similar effect of SO_2_ on tyramine accumulation was evidenced in a model system inoculated with an *O. oeni* strain (Gardini et al., 2005). The control of BA accumulation due to SO_2_ is related to the inhibiting effect on cell metabolism rather than to the repression of the decarboxylases activity (Landete et al., 2008b). Interesting results were obtained in wine using lysozyme instead of SO_2_. In fact, using this enzyme in Rioja wines subjected to malolactic fermentation, a drastic decrease of histamine content was found (López et al., 2009).

## Conclusion

Even if the presence of BAs in food (and the risks associated with them) is known since a long period (Gale, 1946), systematic studies regarding their presence have been carried out only in relatively recent times. The reviews of Shalaby (1996) and Silla Santos (1996) had the merit to collect the fragmented information about this issue and were the starting point for a drastic multiplication of scientific publications regarding the presence of BA in food products and the elucidation of the metabolic and genetic drivers of their production by microorganisms. Combining the words “biogenic amine” and “food” the number of publication selected by the Web of Science passed from about 500 in the year 2000 to more than 4500 in 2015.

This increasing scientific effort allowed obtaining a deeper knowledge about the genetic and biochemical mechanisms responsible for BA production by foodborne microorganisms, but also furnished important information about the possibility to reduce their accumulation in food and the risks associated with their presence.

The possible ways reviewed here to achieve this goal in food are mainly based on two strategies, which always are strictly interacting each other: the modulation of process and environmental factors including storage and distribution conditions and the control of the microbiota associated with fermented foods.

While the studies regarding the genetic bases of microorganism decarboxylating activity have brought to relevant steps forward and new insights on this topic, the role of environmental and technological factors on the overall activity of aminobiogenic microorganisms and on their decarboxylases requires deeper researches aimed to improve the possibility of intervention on the food processes in the perspective of the reduction of the risks associated to the BA presence.

For the fermented foods, from a strictly microbiological point of view, more information is available regarding the possible role of selected starter cultures aimed to overcome and inhibit decarboxylating microbiota. Further work is required to evaluate the real potential and optimize the use of microbial cultures able to degrade BAs and detoxifying them through the action of amino oxidases. In this perspective, also the use of bioprotective cultures producing bacteriocins or other antimicrobial substances needs greater attention due to their not fully explored potential in this field.

## Author Contributions

FG and GS wrote the introduction and conclusion sections; YO and FO wrote chapters 2, 3, 4, and 5; GT coordinated contributions and provided the final draft of the manuscript.

## Conflict of Interest Statement

The authors declare that the research was conducted in the absence of any commercial or financial relationships that could be construed as a potential conflict of interest.
